# Alzheimer's Disease Frontal Cortex Mitochondria Show a Loss of Individual Respiratory Proteins but Preservation of Respiratory Supercomplexes

**DOI:** 10.1155/2019/4814783

**Published:** 2019-03-05

**Authors:** Paula M. Kenney, James P. Bennett

**Affiliations:** Neurodegeneration Therapeutics, Inc., Charlottesville, VA, USA

## Abstract

Alzheimer's disease (AD), the most common cause of sporadic dementia of in adults, shows increased risk of occurrence with aging and is destined to become a major sociomedical tragedy over the next few decades. Although likely complex in origin, sporadic AD is characterized by a progressive and stereotyped neuropathology with aggregated protein deposition (esp beta amyloid (BA) and hyperphosphorylated tau (P-tau)) and neuronal degeneration. To date, prevention of BA synthesis or immune-mediated removal of BA has failed to alter AD progression. Development and testing of P-tau therapeutics are a work in progress. AD brain tissues show multiple system deficits, including loss of respiratory capacity. In the present study there were no differences in mitochondrial mass between AD and CTL samples. We examined mitochondrial preparations of postmortem AD and CTL frontal cortex for relative levels of individual respiratory protein complexes by Western immunoblotting. ANOVA revealed deficiencies of all respiratory complex subunits in AD; post-ANOVA t-testing revealed significant differences in levels of subunits for complexes II, III, and V, borderline significance for subunit of complex IV, and no difference for subunit of complex I. We also examined mitochondrial extracts with blue-native gel electrophoresis combined with immunoblotting for subunits of complexes I and III to search for “respiratory supercomplexes” (RSC's). We found that levels of RSC's did not differ between AD and CTL samples. Mitochondrial preparations from end-stage AD brain tissue showed loss of individual ATP-producing respiration subunits but preservation of levels of assembled respiratory subunits into RSC's. Possible explanations include insufficient sensitivity of our method of RSC detection to find loss of individual subunits, or normal levels of RSC's in AD brain mitochondria coupled with decreased levels of nonassembled respiratory complex subunits. Disease-altering therapies of early AD could include stimulation of mitochondrial biogenesis to overcome loss of respiratory subunits.

## 1. Introduction

Postmitotic tissues such as brain require substantial production of ATP to meet energy requirements. Estimates are that 20-25% of cardiac output, metabolic fuels, and oxygen are consumed by adult brains that typically constitute 2-3% of body weight. Both astrocytes and neurons participate in 2-deoxyglucose uptake by brain, a proxy of brain metabolism [1, 2].

Alzheimer's disease (AD) is a mostly sporadically occurring, aging-related neurodegenerative condition of adults that is characterized in its early stages by brain regional loss of cerebral glucose utilization [3] and increased markers for oxidative stress [4–9]. These two findings suggest impairments of mitochondrial respiration, although other deficits, particularly increasing insulin resistance, may account for some of these changes.

Previously, we [10] and others [4] have shown deficits of mitochondrial respiration in postmortem AD brain. We carried out the present study to investigate the origins of these respiratory deficits in AD brain. In AD brains we found deficits in levels of several individual mitochondrial respiratory subunit proteins. In addition, using blue-native electrophoresis, we observed brain mitochondrial respiratory supercomplexes (RSC's) for complexes I and III that were present at similar levels in both AD and CTL samples. Our findings suggest that AD brain mitochondria have an ATP-producing deficit not reflected in our analysis of RSC levels that could be addressed by approaches to stimulate mitochondrial biogenesis.

## 2. Methods

### 2.1. Tissue Samples

Blocks of slow frozen cortical ribbon from human frontal cortex were obtained from the University of Virginia Brain Resource Facility. These samples were used in our earlier work [11]. Demographics of the brain samples used for RNA sequencing (RNAseq) and mitochondrial preparation and immunoblotting are given in Supplemental File 1. Note that there were no significant differences in subjects' ages for the samples used for RNA preparation. For the mitochondrial immunoblotting study, inadequate CTL tissues were available for samples CTL 147, CTL 151, and CTL 161 used in the RNAseq studies. These CTL samples were substituted by CTL samples 142, 159, and 161. In addition, sample CTL 228 was also added. These substitutions resulted in a significant age difference between AD and CTL (p=0.008).

### 2.2. Preparation of Mitochondria

To isolate a crude mitochondrial fraction, we used similar methods to those described in an earlier publication [10] and were prepared by a modification of the method of Lai and Clark [12] with the Isolation Buffer recommended by Jha, Wang, and Auwerx [13]. 1 gm of slow frozen cortical ribbon from each case was minced, on ice, in 3 ml of Mitochondrial Isolation Buffer (MIB) [200 mM Sucrose, 1 mM Tris Base, and 10 ml/liter 100 mM EGTA/Tris {100 mM EGTA adjusted to pH 7.4 using Tris powder} pH of final solution adjusted to 7.4 with 1 M HEPES and filtered and aliquots were stored at -20°C. 1X Protease Inhibitor Cocktail, Mammalian (VWR, Radnor, PA) added at time of use.]. Minced tissue was homogenized, on ice, for 60 passes in a Dounce homogenizer (0.05 mm clearance) in 7 volumes MIB and centrifuged for 3 min at 1300 X g, 4°C. Supernatants were saved on ice while pellets were homogenized for an additional 30 passes in 6 volumes MIB and centrifuged 3 min at 1300 X g. Supernatants were combined and centrifuged for 80 min at 2,721 X g, 4°C. Pellets were washed with 10 volumes MIB and centrifuged for 80 min at 2,721 X g, 4°C. Final pellet was resuspended in 4 volumes MIB, protein concentrations were determined using a Pierce™ Detergent Compatible Bradford Assay (VWR, Radnor, PA), and 100 ug aliquots were centrifuged 20 min at 17,000 X g, 4°C. Supernatant were aspirated and mitochondrial pellets stored at -80°C.

### 2.3. Sample Preparation for SDS-Page

0.3 g slow frozen human cortical ribbon from each case was homogenized for 30 passes in modified radioimmunoprecipitation assay (RIPA) buffer (50 mM Tris HCl, pH 7.4; NP-40, 0.25% sodium deoxycholate, 150 mM sodium chloride, 1mM PMSF, and 1X Protease Inhibitor Cocktail, Mammalian [VWR, Radnor, PA] added at time of use.). Alternatively, 600 ug of mitochondrial fraction pellets from each case was resuspended in 300 ul RIPA buffer. All RIPA suspensions were sonicated for 4 min in a water bath sonicator, held on ice for 30 min with vortexing every 5 min, and centrifuged at 15,000 X g for 10 min. Supernatants were saved, protein concentrations determined using a Pierce Bradford assay, and 40 ug aliquots stored at -80°C.

### 2.4. SDS-PAGE Immunoblots

BioRad (Hercules, CA) complete XT Sample Buffer was added to 40 ug cortex RIPA lysate or 20 ug mitochondrial RIPA lysate, heated for 5 min at 95°C, loaded on BioRad Criterion XT 4-12% Bis-Tris gels, and electrophoresed for 30 to 40 min 200 v in XT-MES buffer. Gels were equilibrated 10 min in transfer buffer and proteins transferred to PVDF membranes using an Invitrogen iBlot (Life Technologies, Carlsbad, CA) program P3 for 7 min. Transferred membranes were fixed in 8% Acetic Acid, washed with water, and air-dried. Following rehydration (3 x 5 min methanol, 3 x 5 min water), membranes were blocked for 1 hr in Li-Cor Blocking Buffer (Lincoln, NE), incubated 2 hr in primary antibodies, washed, incubated 30 min in secondary antibodies, washed, and imaged on a Li-Cor Odyssey Imager. All primary antibodies were from abcam (Cambridge, MA) and all secondary antibodies were from Li-Cor.

### 2.5. Blue-Native-PAGE Immunoblots

Blue-Native-PAGE of mitochondrial fractions was preformed according to the method of Jha, Wang and Auwerx [13] using a XCell SureLock Mini-Cell, Native Sample Prep Kit, NativePage anode buffer, NuPage Transfer Buffer, and NativeMark Unstained Protein Standard all from Fisher Scientific (Atlanta, GA). Anode, cathode, and sample buffers were prepared, fresh, on the day of use. Briefly, 100 ug mitochondrial pellets were solubilized in 40 ul each 8 g/g digitonin sample buffer for 20 min on ice, centrifuged 10 min at 20,000 X g, 4°C, and 15 ul of supernatant transferred to two fresh tubes. 2 ul Coomassie G250 Sample Additive was added to each tube and prepared samples loaded in wells of Invitrogen NativePage 3-12% Bis-Tris Gels. Gels were run for 25 min at 150 v in Dark Blue Cathode Buffer (0.044 g Coomassie Brilliant Blue G-250 in 220 ml NativePage anode buffer). Dark Blue Cathode Buffer was removed, Light Blue Cathode Buffer (20 ml Dark Blue Cathode Buffer in 180 ml NativePage anode buffer) was added, and gels were run for an additional 3.5 hr at 250 v. Final gels were equilibrated 15 min in NuPage Transfer Buffer and proteins transferred to PVDF membranes using an Invitrogen iBlot program P3 for 14 min. Membranes were washed with water, destained/fixed for 3 x 5 min in 25% Acetic Acid/50% methanol (Liu et al, 2017), washed with water, and air-dried. Following rehydration, membranes were immunolabelled and imaged as above. All primary antibodies were from Abcam (Cambridge, MA) and all secondary antibodies were from Li-Cor.

### 2.6. RNA Sequencing (RNAseq)

Total RNA was extracted from AD and CTL samples indicated in Supplemental File 1, using the Qiagen miRNeasy® system, which yields both mRNA's of all sizes and small, noncoding RNA's that include microRNA's (miRNA's). On column DNAase treatment was carried out for all samples. RNA was quantitated by spectroscopy and analyzed on a BioAnalyzer®. RNA samples were then frozen at -80 degrees and sent on dry ice to Cofactor Genomics (CFG, http://www.cofactorgenomics.com; St. Louis, MO) for ribosomal RNA (rRNA) depletion and construction of Illumina multiplex sequencing libraries. After passing CFG's quality control, sequencing libraries were analyzed on an Illumina® sequencer using paired-end (PE) technology and ~60 million PE reads/sample.

Bioinformatic analyses of gz-compressed PE sequencing fastq files were downloaded from CFG and carried out at Neurodegeneration Therapeutics, Inc., (NTI) on a MacPro desktop. They consisted of examination of each sequencing file with FastQC, removal of Illumina® sequencing adapters with Trimmomatic®, and alignment with HISAT2 [14] (HISAT2 version 2.1.0 release 6/8/2017; https://ccb.jhu.edu/software/hisat2/index.shtml) using the Cufflinks option for SAM file formatting, conversion of SAM files to sorted.BAM files with Samtools and Cufflinks quantitation of gene expression abundance using the latest hg38 genes.gtf file. The script for the HISAT2-Samtools-Cufflinks pipeline is given in Supplemental File 2.

Prism version 7.0a (https://www.graphpad.com) was used for all plotting and statistical analyses.

## 3. Results

**Figure 1:**
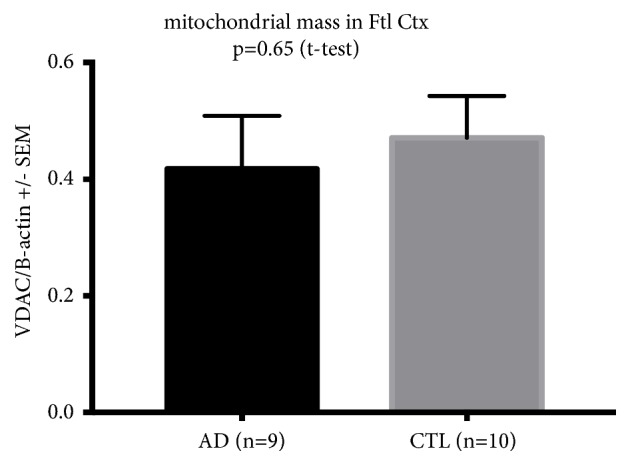
*No differences in apparent mitochondrial mass between CTL and AD frontal cortex samples.* Shown are mean +/- SEM values for beta actin-normalized VDAC/porin levels in frontal cortex samples from CTL and AD whole tissue samples. There were no significant differences between CTL and AD samples.

Figure 1 shows plots of immunoblot results of VDAC (porin) levels normalized to tissue beta actin levels. There were no differences among CTL and AD frontal cortex samples. This result indicates no differences in detectable mitochondrial mass between AD and CTL samples.

**Figure 2:**
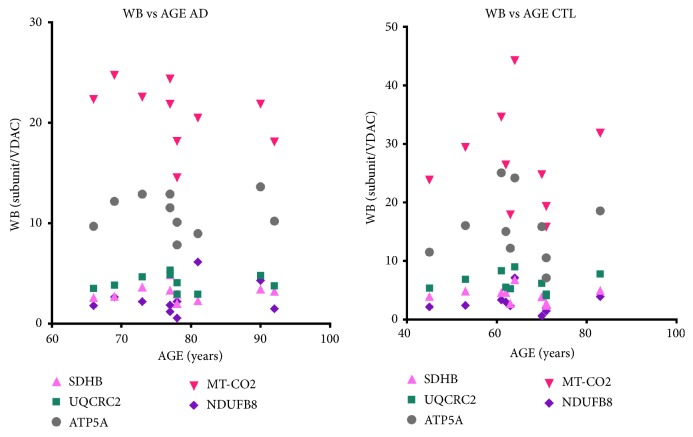
*No relationships among subject ages and levels by immunoblotting of individual respiratory protein subunits.* Shown are relative levels of individual respiratory protein subunits, normalized to mitochondrial mass (VDAC/porin). SDHB= succinate dehydrogenase, subunit B (complex II); UQCRC2= ubiquinol-cytochrome c reductase core protein 2 (complex III); ATP5A = ATP synthase, subunit 5A (complex V); MT-CO2 = mitochondrial DNA-encoded cytochrome oxidase subunit 2 (complex IV); NDUFB8 = NADH: ubiquinone oxidoreductase subunit B8 (complex I). VDAC = voltage-dependent anion channel (abundant mitochondrial outer membrane protein).

Figure 2 shows that there were no relationships among subject age and levels of normalized (to VDAC/porin) individual respiratory protein subunits measured by immunoblotting in both AD and CTL crude mitochondrial samples.

**Figure 3:**
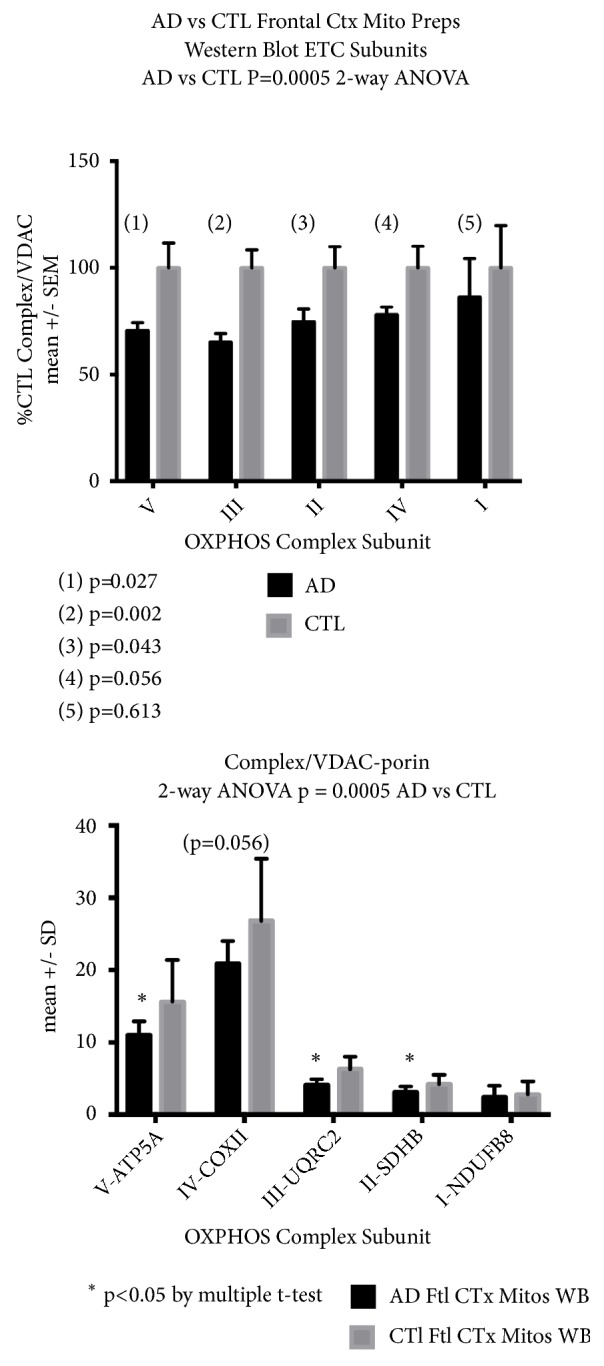
*AD mitochondrial preparations from frontal cortex show reductions in all OXPHOS subunits representative of complexes I-V.* Shown are immunoblot results for mitochondrial preparations of frontal cortex from CTL and AD samples, extracted with RIPA buffer, electrophoresed and immunoblotted as described in Methods, and normalized to mitochondrial mass (VDAC). All OXPHOS subunits showed significant reductions in AD mitochondria (2-way ANOVA), and several subunits showed individual declines in AD brain (t-test). (top) OXPHOS subunit levels are expressed as % mean CTL levels. (bottom) OXPHOS subunit raw data. Abbreviations as in Figure 2.

Figure 3 shows OXPHOS blots of crude mitochondrial preparations, extracted with RIPA buffer, prepared and electrophoresed/blotted as described in* Methods*, and normalized to mitochondrial mass (VDAC/porin). There were significant differences found in reductions in the AD samples, both with regard to all the individual respiratory subunits examined (2-way ANOVA), and multiple individual complex subunits (post-ANOVA t-tests). This result indicates a loss of individual respiratory subunits in the mitochondria. Images of immunoblots for OXPHOS complexes can be found in Supplemental Figure 1.

**Figure 4:**
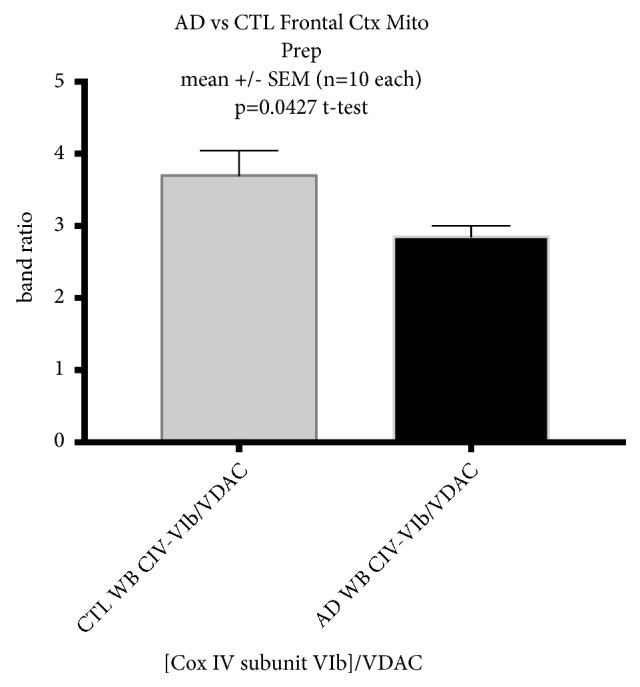
*AD frontal cortex mitochondrial samples show reduction in levels of complex IV subunit 6B protein.* Shown are mean +/- SEM of immunoblot data from frontal cortex mitochondrial preparations of CTL and AD samples, immunostained for the nuclear genome-encoded complex IV subunit 6B.

Figure 4 shows results for immunostaining of complex IV, subunit 6b, which is a nuclear genome-encoded subunit. We carried out this analysis because all of the subunits of our OXPHOS antibody cocktail were made against nuclear genome-encoded subunits, except for complex IV, which was made against subunit 2 that is mtDNA-encoded. We found that VDAC-normalized CIV, subunit 6B levels, were significantly reduced in AD.

**Figure 5:**
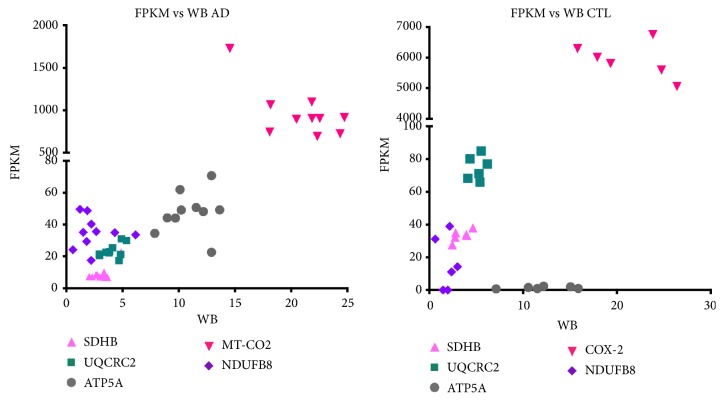
*Relationships among levels of VDAC-normalized OXPHOS subunits in frontal cortex compared to their gene expression values from Cufflinks analysis of RNAseq data.* Total RNA was extracted from AD and CTL frontal cortex samples, subjected to multiplex paired-end RNA sequencing and gene expressions analyzed as described in* Methods*. These expression levels (as FPKM) were plotted against relative levels of their respective respiratory proteins as assayed by Western blotting of mitochondrial preparations from the same samples (see* Methods* for details).

Figure 5 shows the relationships among VDAC-normalized OXPHOS subunits in frontal cortex whole homogenates compared to their gene expression values from Cufflinks analysis of RNAseq data (shown as FPKM (fragments per kilobase per million bases sequenced) generated by Cufflinks following HISAT2 alignment). In general there were no obvious linear relationships between gene expression FPKM values and Western blot signals for the OXPHOS subunits, in both CTL and AD samples.

**Figure 6:**
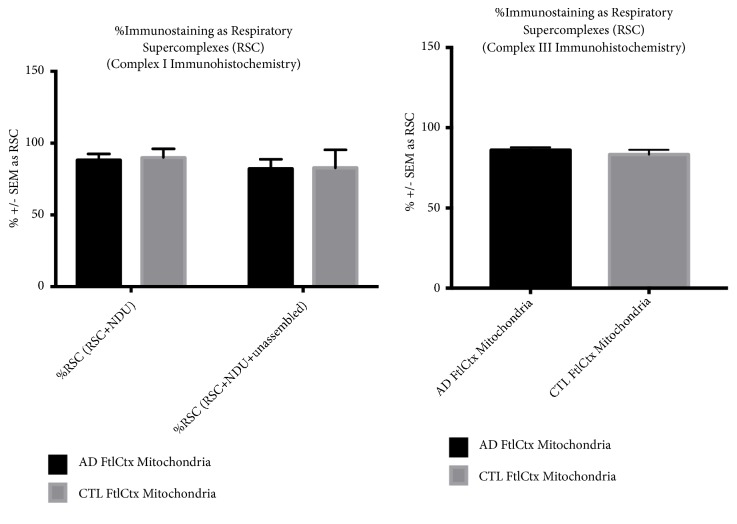
*Quantitative immunoblotting (IB) of respiratory supercomplexes (RSC) isolated by digitonin extraction of crude mitochondrial fractions*. Mitochondrial RSC were separated by blue-native gel electrophoresis as described in* Methods*. Proteins were then transferred to nylon membranes and immunoblotted for complex I (shown in top figure); complex III IB is shown in bottom figure. See* Supplemental Figure *2 for representative immunoblots.

Figure 6 shows CI respiratory supercomplexes (RSC) (top) and CIII RSC (bottom) as % of total CI or CIII. Note that most of the signals for both CI and CIII were found in RSC. Images of RSC can be found in Supplemental Figure 2.

## 4. Discussion

AD is already a major sociomedical problem that will only worsen as more of our population age. To date, no single approach has altered the course of decline in AD, although a multicomponent lifestyle change program has shown promise [15]. These difficulties in achieving success in altering the course of decline in AD likely have multiple origins, including the possibility that AD at the molecular level may be a heterogenous disorder in spite of commonly observed microscopic neuropathological changes.

Because of the brain's disproportionately large energy substrate use, oxygen consumption and ATP requirements, and OXPHOS deficits found in AD brain and other tissues, we [10] and others [16] have conducted multiple studies to determine potential origins of these respiratory deficiencies. AD brain and peripheral tissues demonstrate deficits in electron transport/ATP production and increases in oxidative stress.

There appear to be multiple origins of these respiratory problems. AD mitochondrial DNA is functionally altered, as determined in cybrid studies [9], and shows excessive mutations [9]. AD mitochondria show reduced respiratory capacity and increased oxidative stress damage. One of the earliest detectable changes in AD subjects (who have a clinical precursor state known as “mild cognitive impairment”) is the loss of cerebral glucose metabolism [3], consistent with but not diagnostic of reduced oxidative phosphorylation. These observations suggest that loss of respiratory capacity occurs early in AD pathogenesis and may comprise a focal point of therapeutic intervention.

Using protein immunoblotting we found no reduction in apparent mitochondrial mass in AD frontal cortex brain samples, but we did note reductions in levels of individual respiratory protein subunits in crude mitochondrial subfractions.

There was no apparent decline in levels of respiratory supercomplexes (RSC's), which we detected with blue-native gel electrophoresis. As far as we are aware, ours is the first report of RSC's in postmortem human brain. Prior studies have involved fresh (not frozen) mitochondria isolated from rodent brain [13] or human fibroblasts [17]. Comparing our images with those published, we observed more “smear” of higher MW RSC's in both AD and CTL samples, likely arising from use of frozen tissues and long-term storage of samples.

For some but not most respiratory subunits, there was a correlation between levels of mRNA for that subunit and levels of subunit protein in mitochondrial subfractions. These findings suggest either a loss of transcriptional drive (gene expression) or accelerated degradation of mRNA, such as might occur with microRNA's. Conceivably both processes could be taking place. Further studies are needed to sort out the mechanisms of respiratory subunit protein loss.

There are many limitations in our study. Chief among these is the use of postmortem frozen tissues that have been stored and have significant (but generally unavoidable) postmortem delays. Freeze-thawing is predicted to fracture many cellular organelles, including mitochondria. To analyze the greatest mitochondrial mass in each sample, we used a crude mitochondrial preparation that did not include large cellular debris (removed by low speed centrifugations) or ribosomes (present in supernatant). The specificities of our immunoblot analyses derived from antibodies used, not from anatomical purities of mitochondrial preparations.

Another limitation is our use of advanced/end-stage AD samples. There is significant neurodegeneration in multiple forebrain areas of AD, and it is likely that much of the neuronal mitochondrial pathology is missing from our samples. We may be examining changes of “survivor” neurons, in addition to astroglia, and hope that it will be possible in the future to repeat our study in earlier cases (particularly MCI).

There are multiple biases in RNAseq algorithms and limitations of immunoblot analysis techniques used in this study. Hopefully both will improve over time and allow more sophisticated data to be generated.

Are there therapeutic implications of our findings? One of the most straightforward therapeutic approaches would be to stimulate mitochondrial biogenesis (mitobiogenesis), even though “defective” mitochondria might result. An increase of mitochondrial mass, even if composed of reduced respiratory capacity/unit mitochondrial mass, could increase ATP production to normal (or even above normal) levels. Would increasing “defective” mitos increase oxidative stress? If so, stimulating mitobiogenesis could be combined with antioxidant therapy. It is not clear what therapeutic effect, if any, would accrue in such circumstances, but since the therapeutic index for stimulating mitobiogenesis is favorable, this seems like a worthwhile experiment. Peripheral tissues such as platelets or fibroblasts could serve as markers for effective stimulation of mitobiogenesis in individuals.

## Data Availability

All primary data are the property of Neurodegeneration Therapeutics, Inc., and are available upon request to the corresponding author (James P. Bennett Jr.) after completion of a suitable material transfer agreement (MTA).

## Supplementary Materials

Supplementary 1
* Supplemental File 1.* Demographics of the brain tissue samples for AD (RNAseq and mitochondrial preparation/immunoblotting) and CTL (RNAseq) and CTL (mitochondrial preparation/immunoblotting).

Supplementary 2
* Supplemental File 2. *Text script for HISAT2/Samtools/Cufflinks bioinformatics pipeline.

Supplementary 3
* Supplemental Figure 1.* Shown are representative immunoblots for OXPHOS subunits imaged on the Li-Cor near-infrared laser scanner. Lanes from* left* to* right* are* 1-multicolored MW std; 2-human heart mito CTL; 3-137 CTL; 4-138 AD; 5-142 CTL; 6-144 CTL; 7-150 CTL; 8-156 AD; 9-159 CTL; 10-164 CTL; 11-178 AD; 12-190 AD; 13-multicolored MW std; 14-191 CTL; 15-196 AD; 16-208 AD; 17- 212 AD; 18-213 CTL; 19- 215 AD; 20- 216 CTL; 21- 223 AD; 22-228 CTL; 23-248 AD; 24-multicolored MW std.*

Supplementary 4
* Supplemental Figure 2*. Shown are representative immunoblots for RSC's of CI (TOP) and CIII (BOTTOM) imaged on the Li-Cor near-infrared laser scanner. See* Methods* for details. Lanes from* right* to* left* are* 1- 248 AD; 2- 228 CTL; 3- 223 AD; 4- 216 CTL; 5- 215 AD; 6- 213 CTL; 7- 212 AD; 8- 208 AD; 9- 196 AD; 10- 191 CTL.* Note that lanes for CIII (bottom) are narrower than lanes for CI (top).
