# Supplementary material for: Alzheimer's Disease Frontal Cortex Mitochondria Show a Loss of Individual Respiratory Proteins but Preservation of Respiratory Supercomplexes
Source: Int J Alzheimers Dis. 2019 Mar 5;2019:4814783. doi: 10.1155/2019/4814783 (PMC6425380; doi:10.1155/2019/4814783)
Supplement: Supplementary 1 — Supplemental File 1. Demographics of the brain tissue samples for AD (RNAseq and mitochondrial preparation/immunoblotting) and CTL (RNAseq) and CTL (mitochondrial preparation/immunoblotting). [file 4814783.f1.pdf]

Case	Neuropath Dx	Age	RIN	Braak Staging	CERAD Criteria
**138**	AD	78	4.0		
**156**	AD	77	4.0		
**178**	AD	77	6.9		
**190**	AD	78	4.0		
**196**	AD	66	5.0		AD
**208**	AD	92	5.1		AD
**212**	AD	90	4.3		
**215**	AD	73	4.4	V/VI	AD
**223**	AD	69	4.4		AD
**248**	AD	81	4.8	V/VI	AD
**mean**		**78.1**	**4.7**		
**std dev**		**8.2**	**0.9**		
**ADvsCTL RNAseq p (t-test)**		**0.18**	**0.97**		

RNAseq CTLs

**137**	CTL	63	5	N/A	N/A
**144**	CTL	83	4.9	N/A	N/A
**147**	CTL	53	4.9	N/A	N/A
**151**	CTL	88	4.7	N/A	N/A
**161**	CTL	87	4.6	N/A	N/A
**164**	CTL	70	4	N/A	N/A
**191**	CTL	71	4	N/A	N/A
**150**	CTL	53	5.3	N/A	N/A
**216**	CTL	71		N/A	N/A
**mean**		**71**	**4.7**		
**std dev**		**12.5**	**0.5**		

Mito Prep and mmunoblot CTLs

**137**	CTL	63		N/A	N/A
**144**	CTL	83		N/A	N/A
**142**	CTL	62		N/A	N/A
**159**	CTL	45		N/A	N/A
**213**	CTL	61		N/A	N/A
**164**	CTL	70		N/A	N/A
**191**	CTL	71		N/A	N/A

**150**	CTL	53	N/A	N/A
**216**	CTL	71	N/A	N/A
**228**	CTL	64	N/A	N/A
**mean**		**64**		
**std dev**		**10**		
**ADvsCTL Immunoblot p (t-test)**		0.008		
