# Supplementary material for: Alzheimer's Disease Frontal Cortex Mitochondria Show a Loss of Individual Respiratory Proteins but Preservation of Respiratory Supercomplexes
Source: Int J Alzheimers Dis. 2019 Mar 5;2019:4814783. doi: 10.1155/2019/4814783 (PMC6425380; doi:10.1155/2019/4814783)
Supplement: Supplementary 2 — Supplemental File 2. Text script for HISAT2/Samtools/Cufflinks bioinformatics pipeline. [file 4814783.f2.pdf]

Case	Neuropath Dx	Age	RIN	Braak Staging	CERAD Criteria
**138**	AD	78	4.0		
**156**	AD	77	4.0		
**178**	AD	77	6.9		
**190**	AD	78	4.0		
**196**	AD	66	5.0		AD
**208**	AD	92	5.1		AD
**212**	AD	90	4.3		
**215**	AD	73	4.4	V/VI	AD
**223**	AD	69	4.4		AD
**248**	AD	81	4.8	V/VI	AD
**mean**		**78.1**	**4.7**		
**std dev**		**8.2**	**0.9**		
**ADvsCTL RNAseq p (t-test)**		**0.18**	**0.97**		
RNAseq CTLs					
**137**	CTL	63	5	N/A	N/A
**144**	CTL	83	4.9	N/A	N/A
**147**	CTL	53	4.9	N/A	N/A
**151**	CTL	88	4.7	N/A	N/A
**161**	CTL	87	4.6	N/A	N/A
**164**	CTL	70	4	N/A	N/A
**191**	CTL	71	4	N/A	N/A
**150**	CTL	53	5.3	N/A	N/A
**216**	CTL	71		N/A	N/A
**mean**		**71**	**4.7**		
**std dev**		**12.5**	**0.5**		
Mito Prep and mmunoblot CTLs					
**137**	CTL	63		N/A	N/A
**144**	CTL	83		N/A	N/A
**142**	CTL	62		N/A	N/A
**159**	CTL	45		N/A	N/A
**213**	CTL	61		N/A	N/A
**164**	CTL	70		N/A	N/A
**191**	CTL	71		N/A	N/A

**150**	CTL	53	N/A	N/A
**216**	CTL	71	N/A	N/A
**228**	CTL	64	N/A	N/A
**mean**		**64**		
**std dev**		**10**		
**ADvsCTL Immunoblot p (t-test)**		0.008		

cd <path to trimmed fastq files (can be gz compressed)>
hisat2[1] -p 4 --dta-cufflinks -x <path to genome_tran files> -1 x.forward_paired.fq -2 x.reverse_paired.fq -S <path to HISAT2 output> x.sam
cd <path to x.sam> samtools view -bS x.sam > x.bam
samtools sort -T PREFIX -o x.sorted.bam x.bam
cufflinks -o <path to cufflinks output> -G <path to genes.gtf[3]> genes.gtf --library-type fr-firststrand -p 4 -u -b <path to genome.fa> genome.fa[4] <path to x.sorted.bam> x.sorted.bam

[1] download executable file and genome-tran files from https://ccb.jhu.edu/software/hisat2/index.shtml for Windows, Linux or Mac OSX. Be sure executable file is in $PATH
[2] use latest version of Samtools and be sure file is in $PATH
[3] reference gene transcript file in gtf format
[4] reference genome file in FASTA (.fa or .fna) format

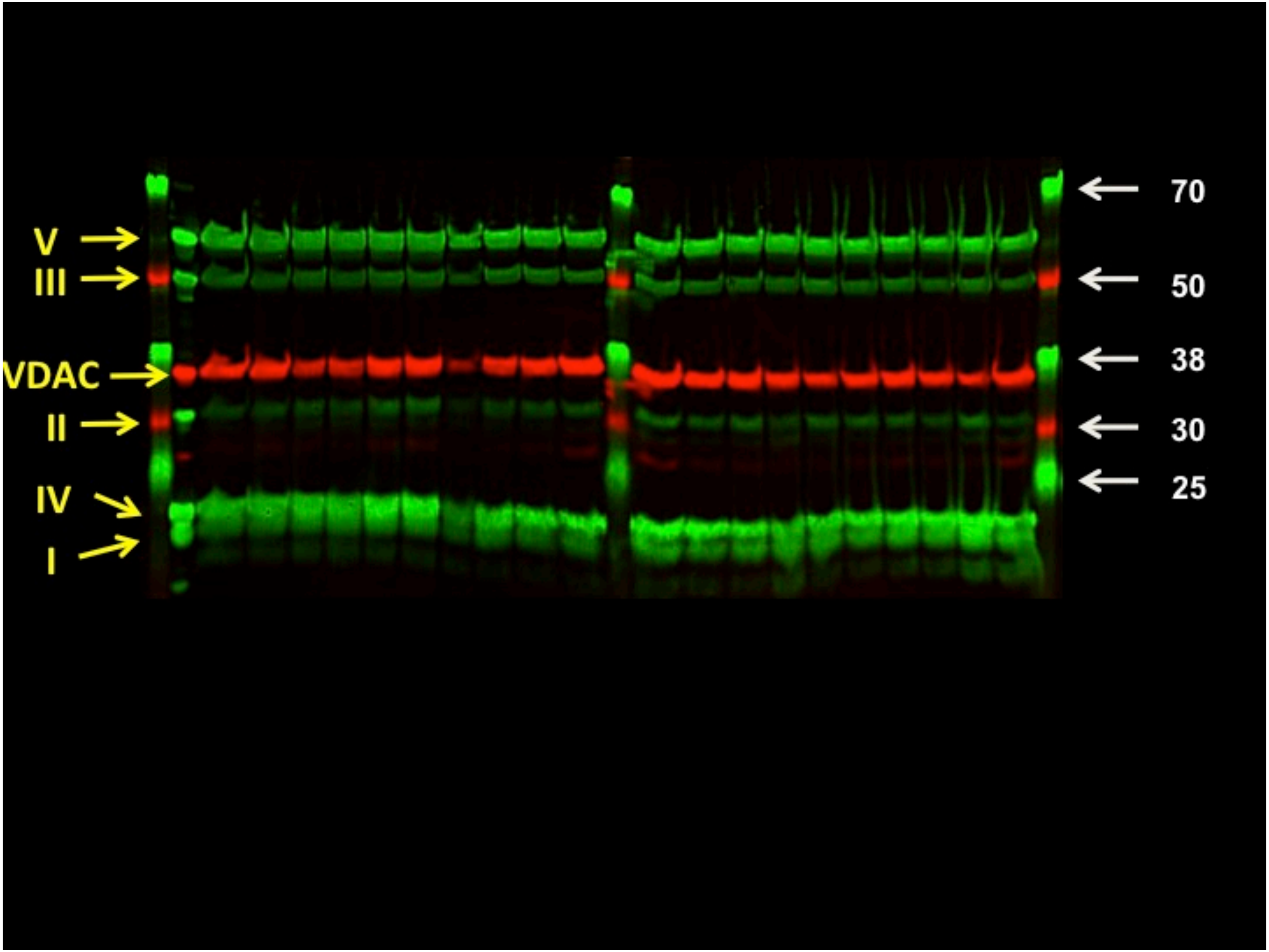
V
III
VDAC
II
IV
I
70
50
38
30
25

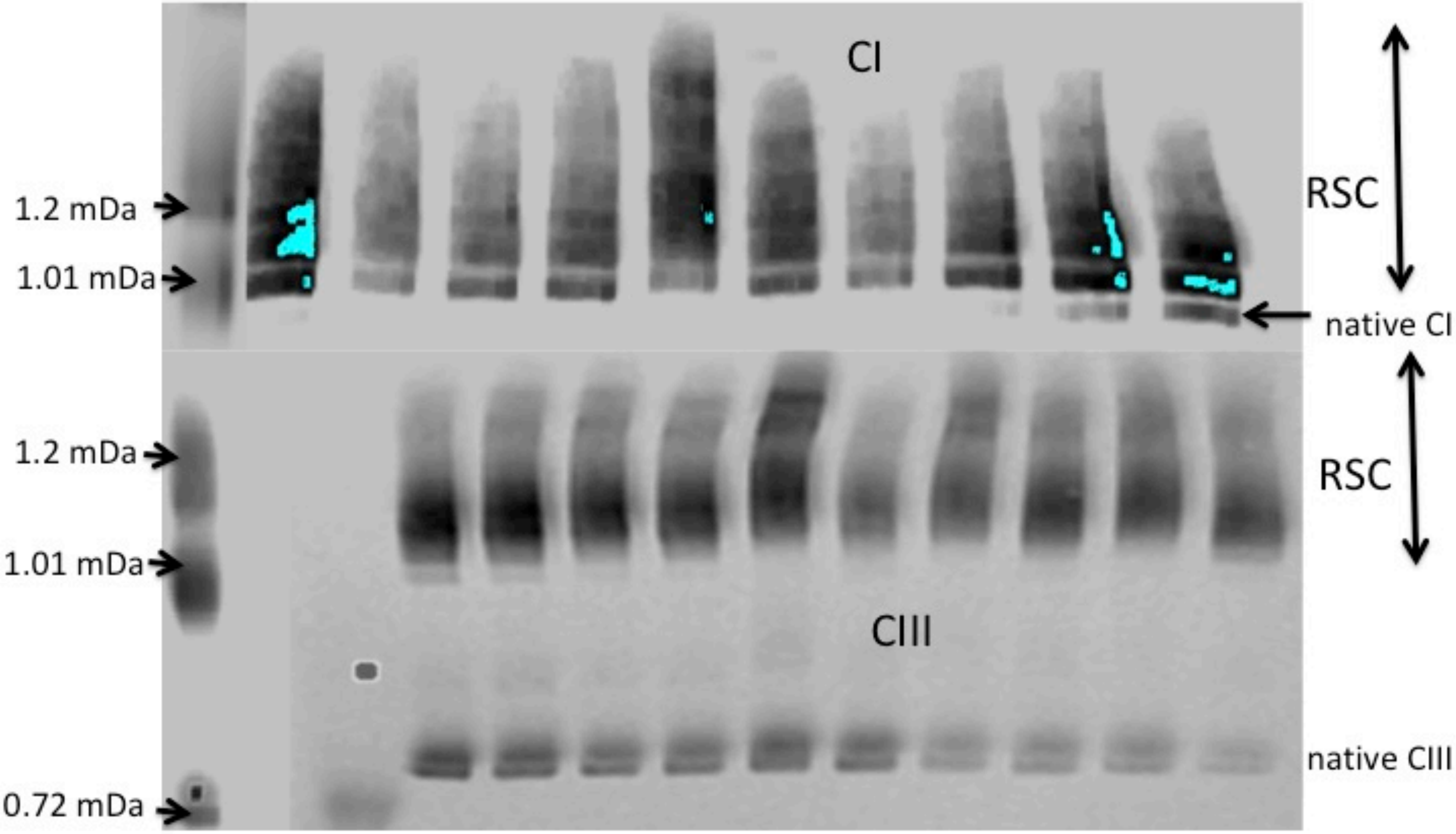
CI
1.2 mDa
1.01 mDa
RSC
native CI
1.2 mDa
1.01 mDa
RSC
CIII
native CIII
0.72 mDa
