# Supplementary figures and images for: Alzheimer's Disease Frontal Cortex Mitochondria Show a Loss of Individual Respiratory Proteins but Preservation of Respiratory Supercomplexes
Source: Int J Alzheimers Dis. 2019 Mar 5;2019:4814783. doi: 10.1155/2019/4814783 (PMC6425380; doi:10.1155/2019/4814783)

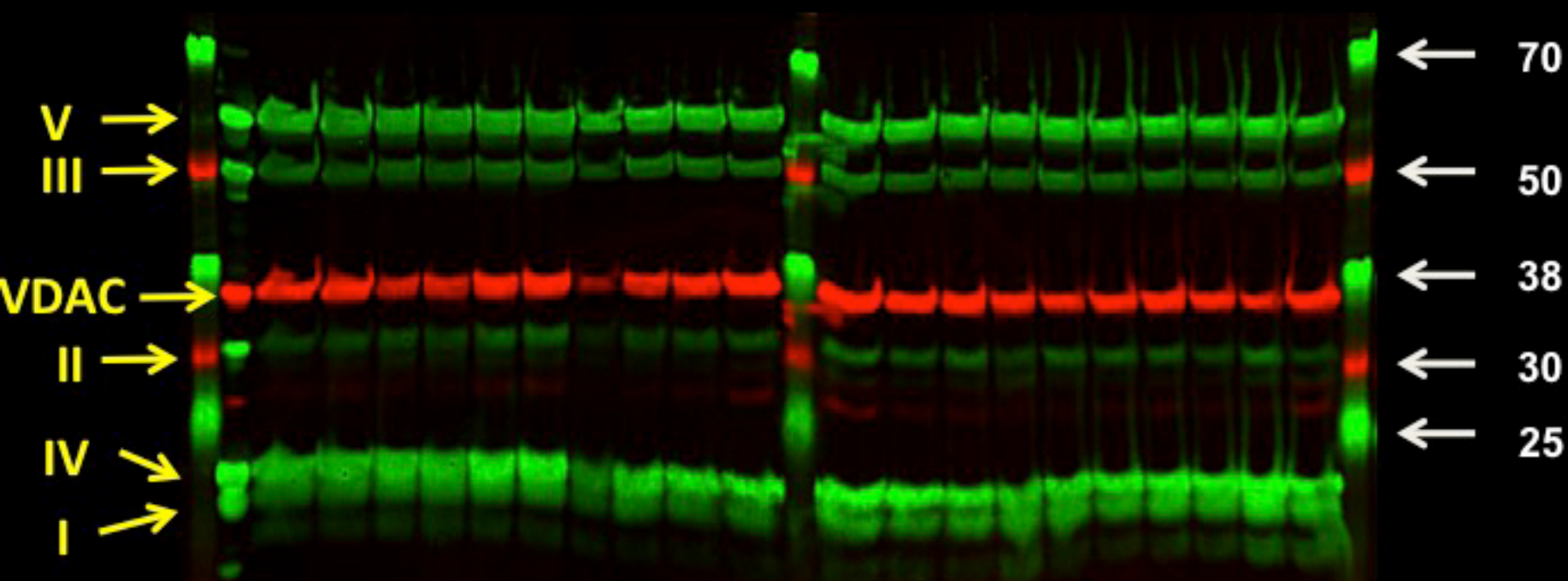

Supplement: Supplementary 3 — Supplemental Figure 1. Shown are representative immunoblots for OXPHOS subunits imaged on the Li-Cor near-infrared laser scanner. Lanes from left to right are 1-multicolored MW std; 2-human heart mito CTL; 3-137 CTL; 4-138 AD; 5-142 CTL; 6-144 CTL; 7-150 CTL; 8-156 AD; 9-159 CTL; 10-164 CTL; 11-178 AD; 12-190 AD; 13-multicolored MW std; 14-191 CTL; 15-196 AD; 16-208 AD; 17- 212 AD; 18-213 CTL; 19- 215 AD; 20- 216 CTL; 21- 223 AD; 22-228 CTL; 23-248 AD; 24-multicolored MW std. [file 4814783.f3.pdf]

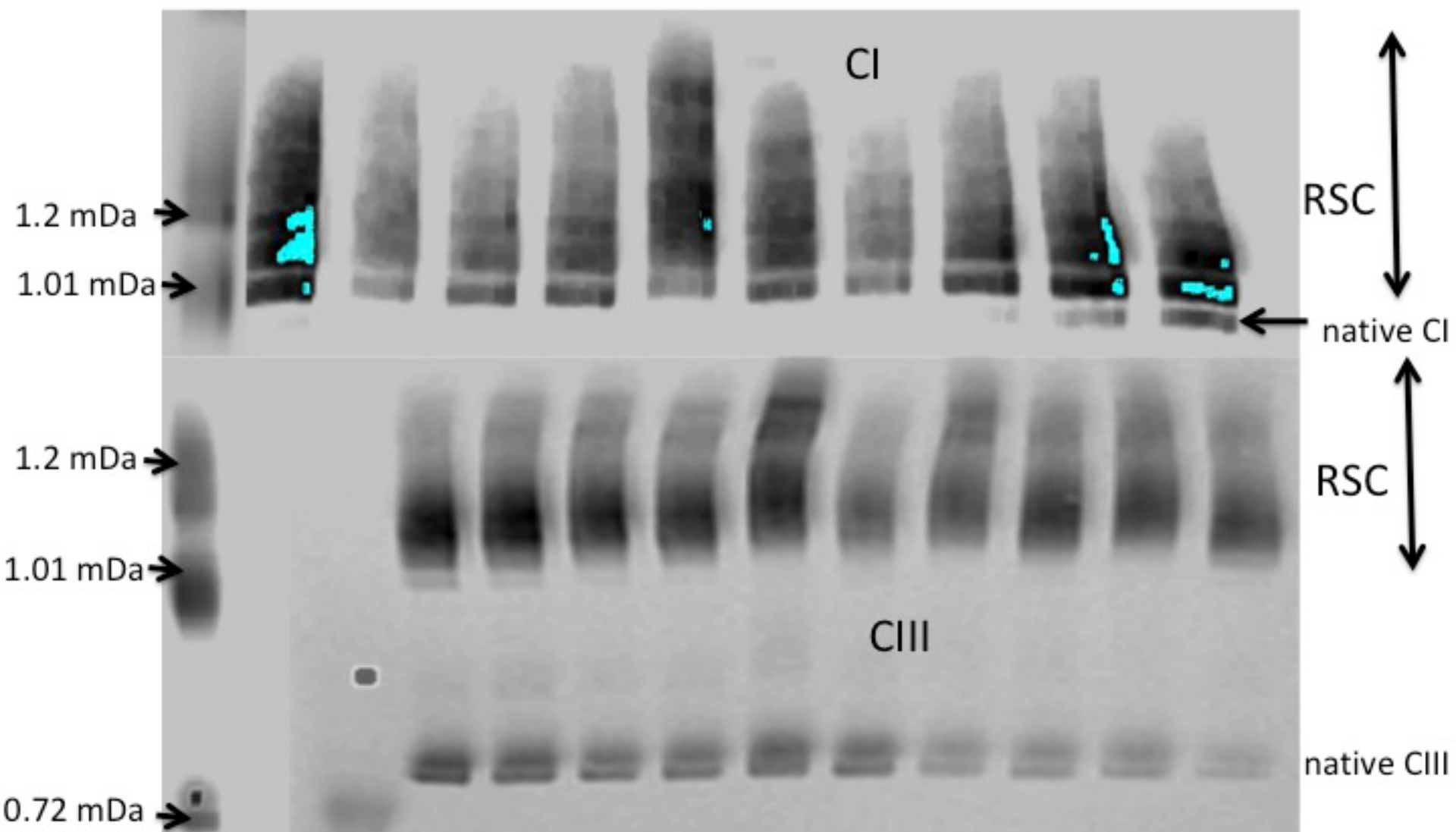

Supplement: Supplementary 4 — Supplemental Figure 2. Shown are representative immunoblots for RSC's of CI (TOP) and CIII (BOTTOM) imaged on the Li-Cor near-infrared laser scanner. See Methods for details. Lanes from right to left are 1- 248 AD; 2- 228 CTL; 3- 223 AD; 4- 216 CTL; 5- 215 AD; 6- 213 CTL; 7- 212 AD; 8- 208 AD; 9- 196 AD; 10- 191 CTL. Note that lanes for CIII (bottom) are narrower than lanes for CI (top). [file 4814783.f4.pdf]
